# L-shaped relationship between atherogenic index of plasma with uric acid levels and hyperuricemia risk

**DOI:** 10.3389/fendo.2024.1461599

**Published:** 2024-12-09

**Authors:** Jingjing Huang, Chunhong Chen, Chunxiao Jie, Ruying Li, Chunyong Chen

**Affiliations:** ^1^ Cardiac Intensive Care Unit, the First Affiliated Hospital of Guangxi Medical University, Nanning, Guangxi, China; ^2^ Department of Endocrinology and Metabolism, National Hospital of Guangxi Zhuang Autonomous Region, Nanning, Guangxi, China; ^3^ Department of Neurology, the First Affiliated Hospital of Guangxi Medical University, Nanning, Guangxi, China

**Keywords:** atherogenic index of plasma, uric acid, hyperuricemia, cross-sectional study, cardiovascular risk

## Abstract

**Background:**

Hyperuricemia is a major risk factor for cardiovascular disease. This study aimed to investigate the relationship between the atherogenic index of plasma (AIP) and serum uric acid (SUA) levels, as well as the risk of hyperuricemia.

**Methods:**

Utilizing data from the National Health and Nutrition Examination Survey (NHANES), we conducted a cross-sectional study involving 9,439 participants aged 18 years and above with complete triglyceride (TG) and high-density lipoprotein cholesterol (HDL-C) data. AIP was calculated as the logarithm of the ratio of TG to HDL-C. Weighted linear regression, weighted logistic regression, subgroup analysis, generalized additive model, restricted cubic spline and two-part linear and logistic regression were utilized to examine the relationships between AIP and SUA levels and hyperuricemia risk.

**Results:**

We identified a non-linear and L-shaped relationship between AIP and both SUA levels and hyperuricemia prevalence, with significant increasing observed up to a saturation point (0.588 for uric acid levels and 0.573 for hyperuricemia prevalence). Below these thresholds, the odds ratios (OR) for increased SUA and hyperuricemia were 0.854 (95% confidence interval [CI]: 0.762, 0.946) and 4.4 (95% CI: 3.528, 5.488), respectively (P<0.001). Beyond these points, the associations were not statistically significant.

**Conclusion:**

Our findings suggest that AIP is significantly and non-linear associated with SUA levels and hyperuricemia risk, with a saturation effect observed beyond specific thresholds. These insights could inform clinical strategies for managing cardiovascular and metabolic risks associated with elevated AIP. Further longitudinal studies are warranted to confirm these associations and elucidate the underlying mechanisms.

## 1 Introduction

Hyperuricemia, characterized by elevated serum uric acid (SUA) levels, is a prevalent metabolic disorder associated with various adverse health outcomes, including gout, cardiovascular disease (CVD), and chronic kidney disease (1, 2). The prevalence of hyperuricemia has been increasing globally, necessitating the identification of novel biomarkers and risk factors to better understand its pathophysiology and improve clinical management (3). Among the potential biomarkers, the atherogenic index of plasma (AIP), defined as the logarithm of the ratio of triglycerides (TG) to high-density lipoprotein cholesterol (HDL-C), has garnered attention due to its association with lipid metabolism and cardiovascular risk (4, 5).

Previous studies have demonstrated that AIP is a reliable predictor of cardiovascular events and metabolic syndrome, suggesting its potential utility in assessing metabolic health (6, 7). However, the relationship between AIP and SUA levels, as well as the risk of hyperuricemia, remains under-explored (8–10). Given the shared metabolic pathways between lipid metabolism and uric acid production, it is plausible that AIP could serve as a valuable marker for hyperuricemia risk stratification (9). Understanding this relationship could provide insights into the underlying mechanisms linking dyslipidemia and hyperuricemia, thereby informing the development of targeted interventions (11, 12).

While previous studies have investigated the relationship between AIP and cardiovascular events and metabolic syndrome (13), the specific patterns of association between AIP and SUA levels and hyperuricemia risk remain incompletely understood. In particular, whether this association exhibits non-linear characteristics and whether there are threshold effects requires further investigation. A deeper understanding of these relationship patterns would not only help elucidate the interaction mechanisms between lipid metabolism and uric acid metabolism but may also provide novel insights for clinical risk assessment.

By employing cross-sectional analysis from the National Health and Nutrition Examination Survey (NHANES), we sought to determine whether AIP is independently associated with SUA levels and hyperuricemia risk after adjusting for potential confounders. Furthermore, we aimed to explore the nonlinear relationship between AIP and SUA levels using restricted cubic spline (RCS) and two-part linear and logistic regression, which allowed for the identification of threshold effects and saturation points. This approach will enable a more nuanced understanding of the AIP-SUA relationship and its implications for hyperuricemia risk. Our findings could potentially inform clinical practice and guide the development of targeted interventions for individuals at a high risk of CVD.

## 2 Materials and methods

### 2.1 Data source

The data used in this study were sourced from the publicly available NHANES database of the United States. All participants provided written informed consent prior to participation (14). The NHANES has a dedicated management system responsible for data collection and updates. The survey data and project information were periodically published on its official the website for public access (14).

### 2.2 Participants

**Figure 1:**
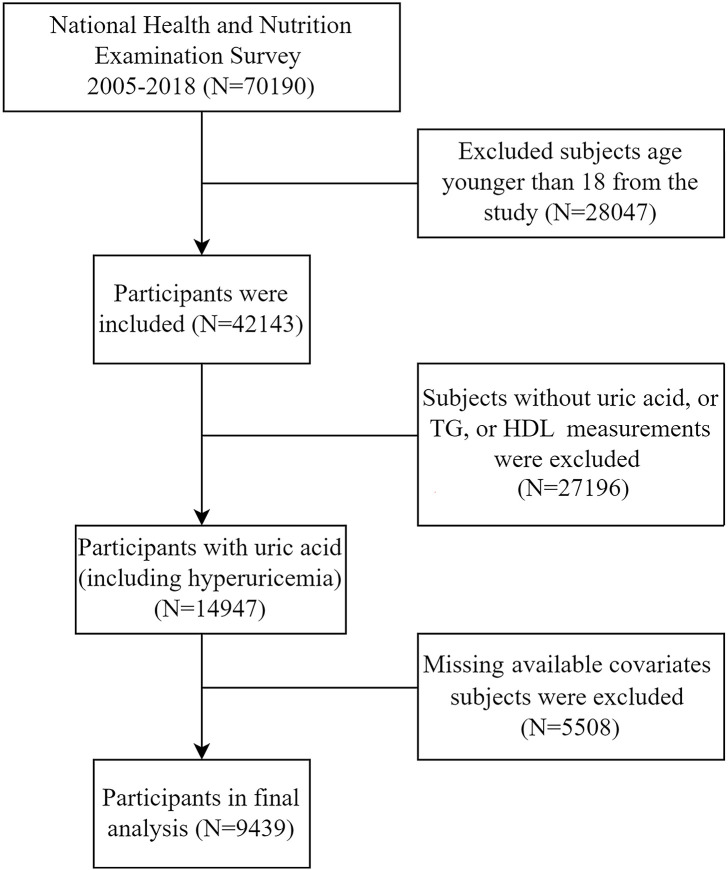
Flowchart depicting the selection process of samples from NHANES during the years 2005 to 2018. TG, triglycerides; HDL-C, high-density lipoprotein cholesterol.

We extracted data on TG, HDL-C, non-HDL-C, SUA, serum creatinine, glycated hemoglobin (HbA1c), urinary protein, urinary albumin, urinary creatinine, and relevant demographic information from the NHANES database. All participants or their guardians had provided informed consent forms prior to participation. Participants were drawn from the NHANES surveys conducted between 2005 and 2018, with an initial total of 70,190 participants. First, 28,047 participants under the age of 18 years were excluded. Additionally, 27,196 participants with missing data on uric acid,TG, or HDL cholesterol were excluded, as well as 5,508 participants with missing relevant covariates. After these exclusions, a total of 9,439 participants were included in the final analysis (**Figure 1**).

### 2.3 Assessment of serum urate and definition of hyperuricemia

SUA levels were measured using a colorimetric assay on a Beckman Synchron LX20 analyzer (Beckman Coulter, Inc., Brea, CA, USA). Hyperuricemia is defined as serum urate >7.0 mg/dL (men) or >5.7 mg/dL (women) (15).

### 2.4 Covariates

This study also assessed the following potential confounding factors: age, sex, race (Mexican American, other Hispanic, non-Hispanic White, non-Hispanic Black, and non-Hispanic Native American), marital status (married/cohabiting, widowed/divorced/separated, and never married), education level (less than 9th grade, 9th-11th grade, high school graduate/GED, some college or AA degree, college graduate or above), alcohol consumption status, smoking status, family poverty income ratio, body mass index (BMI), hypertension status, diabetes status (yes, borderline, and no), serum creatinine, coronary artery disease, heart failure, stroke, sedentary time, HbA1c, total cholesterol (TC), HDL-C, urinary creatinine, and urinary albumin.

### 2.5 Statistical analysis

Statistical analyses were performed utilizing R software (version 4.4.0; R Foundation for Statistical Computing, Vienna, Austria; https://www.r-project.org). The analysis pipeline incorporated specialized R packages for data visualization and statistical modeling (gtsummary, survey, rms, ggplot2, and forestplot). Statistical significance was determined for *P* values less than or equal to 0.05. To account for variability in the dataset, a weight adjustment approach was employed. Participant demographic characteristics were examined through chi-squared tests and Student t tests stratified by uric acid level and hyperuricemia status. Weighted linear regression and logistic regression models were utilized to investigate the relationship between AIP and both uric acid level and hyperuricemia status. AIP was categorized into quartiles as a categorical variable. Trend tests were then applied to assess the linear trend between AIP levels and both uric acid level and hyperuricemia status. Model 1 was an univariable analysis. Model 2 was adjusted for age, sex, and race. Model 3 was adjusted for age, sex, race, family poverty income ratio, BMI, waist circumference, education level, alcohol consumption status, smoking status, marital status, diabetes, hypertension, serum creatinine, coronary artery disease, heart failure, stroke, sedentary time, glycated hemoglobin, urinary albumin/creatinine ratio, urinary creatinine, and urinary albumin.

## 3 Results

### 3.1 Participants characteristics

This study included a total of 9,439 adult participants, who were divided into four quartile groups based on their AIP values (Q1: n = 2,333, Q2: n = 2,352, Q3: n = 2,433, Q4: n = 2,321). We compared the demographic and clinical characteristics among these groups. There were significant differences among the groups in terms of age, sex, family poverty income ratio, race distribution, BMI, waist circumference, education level, smoking status, marital status, diabetes, hypertension, heart failure, HbA1c, serum creatinine, urinary creatinine,TG, HDL-C, TC, HDL-C, non-HDL-C, uric acid, and hyperuricemia indicators (*P* < 0.001). Additionally, coronary heart disease (*P* = 0.004), sedentary time (*P* = 0.015), and urinary albumin/creatinine ratio (*P* = 0.002) also showed significant differences among the groups. Overall, AIP was significantly associated with demographic characteristics, lifestyle, and health indicators in adults. With the increase in AIP values, indicators such as age, BMI, waist circumference, family poverty income ratio, diabetes, hypertension, coronary heart disease, heart failure, SUA, hyperuricemia,TG, TC, and HDL-C showed an increasing trend, while education level and HDL-C levels showed a decreasing trend. (See **Table 1** for detailed baseline characteristic information).

**Table 1:** Characteristics of the population from NHANES 2007-2018.

Characteristic	Q1, N = 2,333	Q2, N = 2,352	Q3, N = 2,433	Q4, N = 2,321	*P*-value
**Age (years)**	45 (30,61)	47 (33,61)	49 (35,63)	49 (37,61)	<0.001
**Age group**					<0.001
20-39 years	904 (40%)	756 (35%)	661 (31%)	601 (26%)	
40-59 years	652 (30%)	701 (32%)	799 (35%)	862 (41%)	
60+ years	777 (30%)	895 (33%)	973 (34%)	858 (32%)	
**Sex**					<0.001
Female	1,494 (63%)	1,318 (57%)	1,185 (49%)	873 (36%)	
Male	839 (37%)	1,034 (43%)	1,248 (51%)	1,448 (64%)	
**Family poverty income ratio**	3.25 (1.59, 5.00)	3.02 (1.52,5.00)	2.75 (1.36,4.94)	2.60 (1.30,4.62)	<0.001
**Race**					<0.001
Mexican American	195 (5.5%)	292 (7.7%)	382 (10%)	403 (11%)	
Other Hispanic	177 (5.2%)	229 (5.8%)	302 (7.3%)	296 (7.1%)	
Non-Hispanic White	810 (64%)	864 (67%)	923 (65%)	1,007 (69%)	
Non-Hispanic Black	742 (16%)	591 (12%)	416 (8.3%)	245 (5.0%)	
Other/multiracial	409 (9.3%)	376 (8.0%	410 (9.5%	370 (8.4%)	
**BMI**	25 (22,29)	28 (24,32)	29 (26,34)	31 (27,35)	<0.001
**BMI group**					<0.001
Normal (18.5 to <25)	1,057 (48%)	731 (30%)	499 (20%)	287 (10%)	
Obese (30 or greater)	519 (19%)	792 (34%)	1,057 (44%)	1,224 (56%)	
Overweight (25 to <30)	657 (29%)	763 (34%)	819 (34%)	771 (33%)	
Underweight (<18.5)	82 (3.3%)	43 (1.6%)	25 (0.8%)	11 (0.6%)	
**Waist Circumference (cm)**	89 (80,98)	96 (87,107)	101 (92,112)	107 (98,117)	<0.001
**Education level**					<0.001
Less than high school	146 (3.6%)	224 (5.0%)	237 (5.2%)	265 (6.5%)	
High school or equivalent	716 (27%)	804 (31%)	903 (34%)	871 (36%)	
More than high school	1.471 (69%)	1.324 (64%)	1.293 (60%)	1,185 (57%)	
**Alcohol consumption status**					0.8
Drinker	1,101 (78%)	1,144 (77%)	1,169 (77%)	1,220 (78%)	
Non-drinker	431 (22%)	468 (23%)	502 (23%)	433 (22%)	
**Smoke group**					<0.001
Current smoker	360 (15%)	403 (15%)	489 (21%)	569 (24%)	
Former smoker	501 (24%)	522 (23%)	607 (26%)	630 (29%)	
Never smoker	1,472 (61%)	1,427 (62%)	1,337 (53%)	1,122 (47%)	
**Marriage group**					<0.001
Married/Living with partner	1,280 (61%)	1,383 (63%)	1,515 (64%)	1,493 (67%)	
Widowed/Divorced/Separate	463 (16%)	532 (19%)	521 (18%)	506 (19%)	
Never married	590 (22%	437 (18%)	397 (17%)	322 (14%)	
**Diabetes**					<0.001
Yes	148 (3.7%)	263 (7.3%)	412 (12%)	503 (19%)	
No	2.125 (94%)	2,029 (91%	1,960 (86%)	1.745 (78%)	
Borderline	60 (2.1%)	60 (2.1%)	61 (2.2%)	73 (3.1%)	
**Hypertension**	682 (24%)	831 (31%)	967 (37%)	1,047 (44%)	<0.001
**Coronary heart disease**	64 (2.7%)	84 (2.8%)	119 (3.9%)	124 (5.1%)	0.004
**Heart failure**	52 (1.5%)	73 (2.2%)	84 (2.6%)	123 (4.6%)	<0.001
**Stroke**	77 (2.6%)	89 (2.8%)	94 (3.1%)	98 (3.6%)	0.3
**Sedentary time (hour)**	6.0 (4.0,8.0)	6.0 (4.0,8.0)	6.0 (4.0,8.0)	6.0 (4.0,9.0)	0.015
Sedentary group					0.3
<3 hours	342 (13%)	311 (11%)	341 (11%)	291 (10%)	
>6 hours	897 (41%)	969 (44%)	983 (44%)	990 (45%)	
3-6 hours	1,094 (46%)	1,072 (45%)	1,109 (45%)	1,040 (45%)	
**Serum creatinine (mg/dL)**	72 (61,83)	72 (62,86)	76 (64,88)	78 (65,89)	<0.001
**HbA1c (%)**	5.30 (5.10,5.60)	5.40 (5.20,5.70)	5.50 (5.20,5.80)	5.60 (5.30,6.10)	<0.001
**uACR (mg/g)**	7 (4,12)	6 (4,11)	7 (4,12)	7 (5,15)	0.002
**Urinary creatinine (mg/dL)**	105 (56,160)	108 (63,162)	114 (70,173)	120 (73,173)	<0.001
**Urinary albumin (ug/mL)**	7 (4,14)	7 (4,13)	8 (4,17)	9 (5,19)	<0.001
**TC (mmol/L)**	4.64 (4.03,5.25)	4.76 (4.16,5.43)	4.89 (4.19,5.59)	5.17 (4.47,5.97)	<0.001
**TG (mmol/L)**	0.59 (0.47,0.70)	0.91 (0.79, 1.06)	1.30 (1.13,1.50)	2.17 (1.80,2.81)	<0.001
**LDL-C (mmol/L)**	2.53 (2.07,3.10)	2.85 (2.33,3.44)	2.97 (2.41,3.62)	3.05 (2.41, 3.72)	<0.001
**HDL-C (mmol/L)**	1.78 (1.53,2.02)	1.45 (1.27,1.66)	1.24 (1.11,1.40)	1.03 (0.91, 1.16)	<0.001
**non-HDL-C**	2.79 (2.33,3.39)	3.29 (2.74,3.88)	3.59 (3.00,4.24)	4.14 (3.44,4.89)	<0.001
**AIP**	-0.46 (-0.58, -0.38)	-0.20 (-0.26,-0.14)	0.02 (-0.04, 0.07)	0.30 (0.22,0.44)	<0.001
**Uric acid (mg/dL)**	4.80 (4.00,5.70)	5.20 (4.30,6.00)	5.60 (4.70,6.50)	6.01 (5.10,6.90)	<0.001
**Hyperuricemia**	276 (11%)	423 (16%)	600 (24%)	787 (34%)	<0.001

AIP, atherogenic index of plasma; BMI, body mass index; HbA1c, Hemoglobin A1c; HDL-C, High-density lipoprotein cholesterol; HTN, hypertension; LDL-C, low-density lipoprotein-cholesterol; TC, total cholesterol; TG, triglyceride; uACR, urine albumin/urine creatinine; OR, odds ratio.

### 3.2 Weighted regression analysis of AIP’s associations with uric acid and hyperuricemia in adults

**Figure 2:**
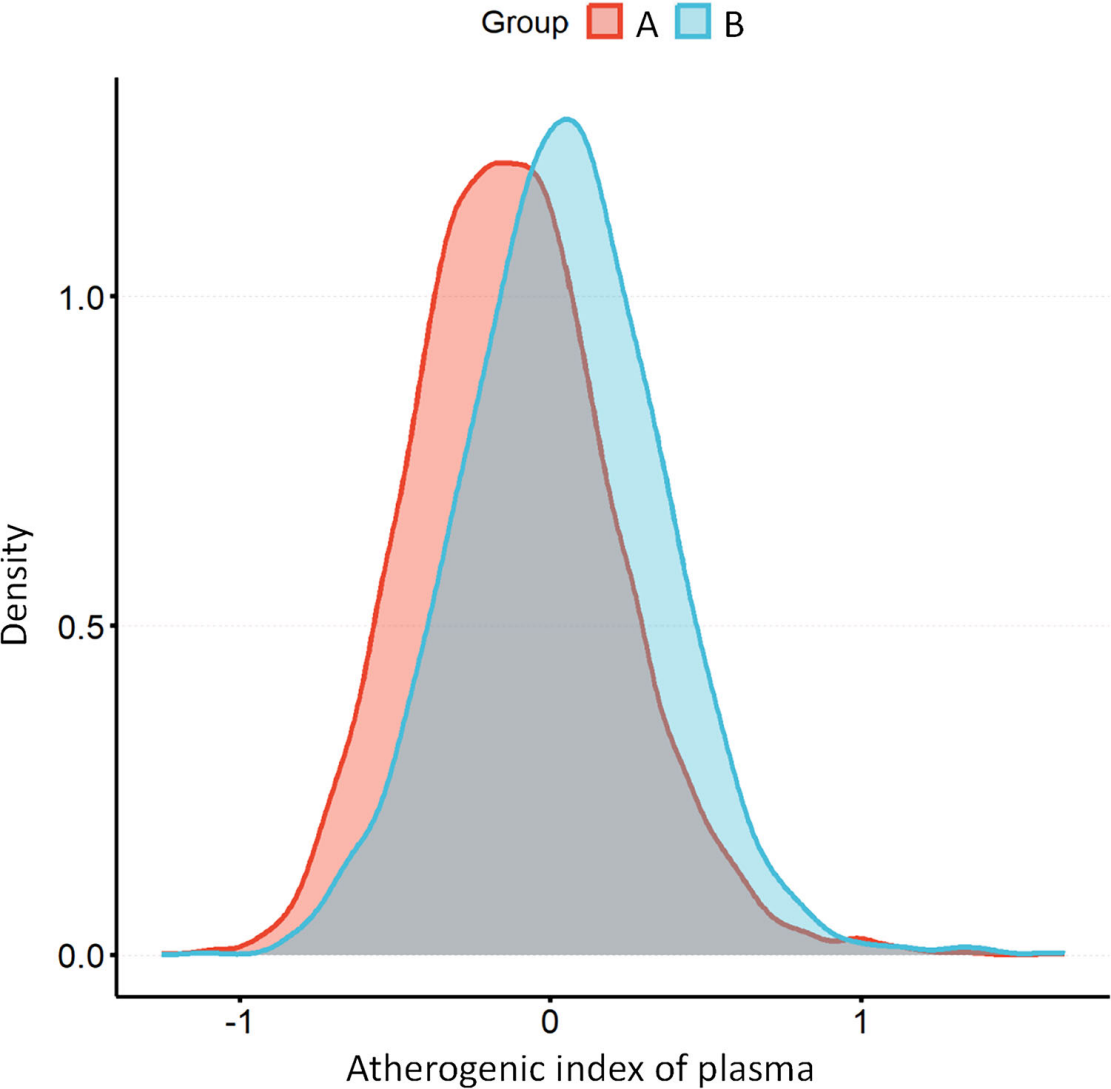
The distribution of AIP among individuals without hyperuricemia **(A)** compared to those with hyperuricemia **(B)**. AIP, atherogenic index of plasma.

**Figure 2** shows the overall distribution of AIP in individuals with and without hyperuricemia. Weighted regression associations between AIP and uric acid levels as well as hyperuricemia in adults are presented in the **Table 2**. In the analysis of uric acid levels, AIP as a continuous variable demonstrated a significant positive correlation across all models (**Table 3**): in Model 1, the β value was 1.3 (95% CI: 1.2-1.5, *P* < 0.001); in Model 2, the β value was 1.0 (95% CI: 0.87-1.1, *P* < 0.001); and in Model 3, the β value was 0.6 (95% CI: 0.43-0.78, *P* < 0.001). When AIP was categorized into quartiles, the associations remained significant: for Q2, the β values were 0.37 (95% CI: 0.28-0.46, *P* < 0.001) in Model 1, 0.31 (95% CI: 0.23-0.39, *P* < 0.001) in Model 2, and 0.13 (95% CI: 0.02-0.23, *P* = 0.021) in Model 3; for Q3, the β values were 0.72 (95% CI: 0.62-0.83, *P* < 0.001), 0.58 (95% CI: 0.48-0.68, *P* < 0.001), and 0.29 (95% CI: 0.18-0.39, *P* < 0.001) in Models 1, 2, and 3, respectively; and for Q4, the β values were 1.2 (95% CI: 1.1-1.3, *P* < 0.001), 0.94 (95% CI: 0.83-1.1, *P* < 0.001), and 0.54 (95% CI: 0.37-0.71, *P* < 0.001) in Models 1, 2, and 3, respectively. The trend across quartiles was statistically significant in all models (*P* for trend < 0.001).

**Table 2:** Weighted regression association of AIP with uric acid and hyperuricemia in adults.

	Model 1		Model 2		Model 3	
	β (95%CI)	*P*	β (95%CI)	*P*	β (95%CI)	*P*
Uric acid						
AIP, continues	1.3 (1.2,1.5)	<0.001	1.0 (0.87,1.1)	<0.001	0.6 (0.43,0.78)	<0.001
AIP, Quartile						
Quartile 1	Reference		Reference		Reference	
Quartile 2	0.37 (0.28,0.46)	<0.001	0.31 (0.23,0.39)	<0.001	0.13 (0.02,0.23)	0.021
Quartile 3	0.72 (0.62,0.83)	<0.001	0.58 (0.48,0.68)	<0.001	0.29 (0.18,0.39)	<0.001
Quartile 4	1.2 (1.1,1.3)	<0.001	0.94 (0.83,1.1)	<0.001	0.54 (0.37,0.71)	<0.001
*P* for trend	<0.001		<0.001		<0.001	
Hyperuricemia						
AIP, continues	4.29 (3.35,5.49)	<0.001	5.13 (3.93,6.71)	<0.001	3.04 (1.93,4.79)	<0.001
AIP, Quartile						
Quartile 1	Reference		Reference		Reference	
Quartile 2	1.61 (1.23,2.11)	<0.001	1.67 (1.26,2.21)	<0.001	1.42 (1.09,2.25)	0.013
Quartile 3	2.57 (1.99,3.32)	<0.001	2.78 (2.13,3.63)	<0.001	1.94 (1.34,2.81)	0.003
Quartile 4	4.13 (3.17,5.38)	<0.001	4.76 (3.59,6.31)	<0.001	2.62 (1.62,4.23)	0.001
*P* for trend	<0.001		<0.001		<0.001	

AIP, atherogenic index of plasma; BMI, body mass index; HTN, hypertension; LDL-C, low-density lipoprotein-cholesterol; TC, total cholesterol; OR, odds ratio. Model 1: univariable; Model 2: age, sex, race; Model 3: age, sex, race, family poverty income ratio, BMI, waist circumference, education level, alcohol consumption status, smoking status, marital status, diabetes, HTN, serum creatinine, coronary heart disease, heart failure, stroke, sedentary time, LDL-C, TC, glycohemoglobin, urine albumin/urine creatinine, urinary creatinine, urinary albumin.

**Table 3:** Saturation effect analysis of AIP on uric acid levels and the prevalence of hyperuricemia.

	Model 1		Model 2		Model 3	
	β(95%CI)	*P*	β(95%CI)	*P*	β(95%CI)	*P*
Uric acid						
Inflection point(K)	0.578		0.571		0.588	
<K effect size OR(95%CI)	1.438 (1.345,1.531)	<0.001	1.197(1.108,1.285)	<0.001	0.854(0.762,0.946)	<0.001
>K effect size OR(95%CI)	-1.088 (-2.24,0.065)	0.064	-0.849(-1.959,0.26)		-0.893(-0.2101,0.316)	0.147
*P* for log likelihood ratio test	<0.001		<0.001		<0.001	
Hyperuricemia						
Inflection point(K)	0.524		0.547		0.573	
<K effect size OR(95%CI)	5.587 (4.652,6.712)	<0.001	7.496 (6.176,9.099)	<0.001	4.4 (3.528,5.488)	<0.001
>K effect size OR(95%CI)	0.311 (0.082,1.182)	0.086	0.489 (0.109,2.191)	0.349	0.313 (0.044,2.242)	0.247
*P* for log likelihood ratio test	<0.001		<0.001		<0.001	

AIP, atherogenic index of plasma; BMI, body mass index; HTN, hypertension; LDL-C, low-density lipoprotein-cholesterol; TC, total cholesterol; OR, odds ratio. Model 1: univariable;Model 2: age,sex, race; Model 3: age, sex, race, family poverty income ratio, BMI, waist circumference, education level, alcohol consumption status, smoking status, marital status, diabetes, HTN, serum creatinine, coronary heart disease, heart failure, stroke, sedentary time, LDL-C, TC, glycohemoglobin, urine albumin/urine creatinine, urinary creatinine, urinary albumin.

In the analysis of hyperuricemia, AIP as a continuous variable also showed significant positive correlations across all models (**Table 3**): in Model 1, the β value was 4.29 (95% CI: 3.35-5.49, *P* < 0.001); in Model 2, the β value was 5.13 (95% CI: 3.93-6.71, *P* < 0.001); and in Model 3, the β value was 3.04 (95% CI: 1.93-4.79, *P* < 0.001). When AIP was categorized into quartiles, the associations remained significant: for Q2, the β values were 1.61 (95% CI: 1.23-2.11, *P* < 0.001) in Model 1, 1.67 (95% CI: 1.26-2.21, *P* < 0.001) in Model 2, and 1.42 (95% CI: 1.09-2.25, *P* = 0.013) in Model 3; for Q3, the β values were 2.57 (95% CI: 1.99-3.32, *P* < 0.001), 2.78 (95% CI: 2.13-3.63, *P* < 0.001), and 1.94 (95% CI: 1.34-2.81, P = 0.003) in Models 1, 2, and 3, respectively; and for Q4, the β values were 4.13 (95% CI: 3.17-5.38, *P* < 0.001), 4.76 (95% CI: 3.59-6.31, *P* < 0.001), and 2.62 (95% CI: 1.62-4.23, *P* = 0.001) in Models 1, 2, and 3, respectively. The trend across quartiles was statistically significant in all models (*P* for trend < 0.001).

### 3.3 Assessing the linkage between AIP and the prevalence of hyperuricemia and elevated uric acid levels

**Figure 3:**
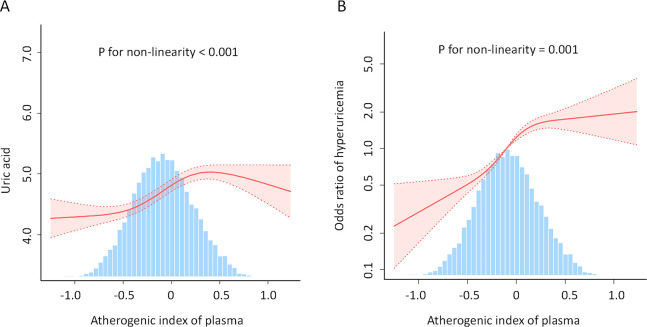
Association between AIP and uric acid levels **(A)** and hyperuricemia **(B)**. An L-shaped, non-linear relationship was observed between AIP and both uric acid levels and the prevalence of hyperuricemia (P < 0.05). The solid line depicts the estimated values, while the dashed line represents the corresponding 95% confidence interval. The inflection points were 0.588 **(A)** and 0.573 **(B)**. Adjustment factors included age, sex, race, family poverty income ratio, BMI, waist circumference, education level, alcohol consumption status, smoking status, marital status, diabetes, hypertension, serum creatinine, coronary heart disease, heart failure, stroke, sedentary time, low-density lipoprotein-cholesterol, total cholesterol; glycohemoglobin, urine albumin/urine creatinine, urinary creatinine, and urinary albumin. AIP, atherogenic index of plasma; BMI, body mass index.

Here, we performed a RCS to detect the non-linear relationships of AIP with uric acid levels and the risk of hyperuricemia and to further confirm the results. In the fully adjusted mode, a non-linear and reverse L-shaped association was detected between AIP and uric acid levels and hyperuricemia (**Figure 3**). The two-part linear and logistic regression analyses of the AIP for uric acid levels and hyperuricemia are presented in **Table 3**. For uric acid levels, the AIP thresholds (K) in models 1, 2, and 3 were 0.578, 0.571, and 0.588, respectively. Below the thresholds, there was a significant positive correlation (Model 1: OR=1.438, 95% CI: 1.345-1.531, *P* < 0.001; Model 2: OR=1.197, 95% CI: 1.108-1.285, *P* < 0.001; Model 3: OR=0.854, 95% CI: 0.762-0.946, *P* < 0.001). Above the thresholds, the effect was not statistically significant (Model 1: *P*=0.064; Models 2 and 3: *P* > 0.05). The log-likelihood ratio test results were significant across all models (*P* < 0.001). For hyperuricemia, the AIP thresholds (K) in models 1, 2, and 3 were 0.524, 0.547, and 0.573, respectively. Below the thresholds, there was a significant positive correlation (Model 1: OR=5.587, 95% CI: 4.652-6.712, *P* < 0.001; Model 2: OR=7.496, 95% CI: 6.176-9.099, *P* < 0.001; Model 3: OR=4.4, 95% CI: 3.528-5.488, *P* < 0.001). Above the thresholds, the effect was not statistically significant (all models *P* > 0.05). The log-likelihood ratio test results were significant across all models (*P* < 0.001).

### 3.4 Examining the subgroup-specific impacts of AIP and hyperuricemia

**Figure 4:**
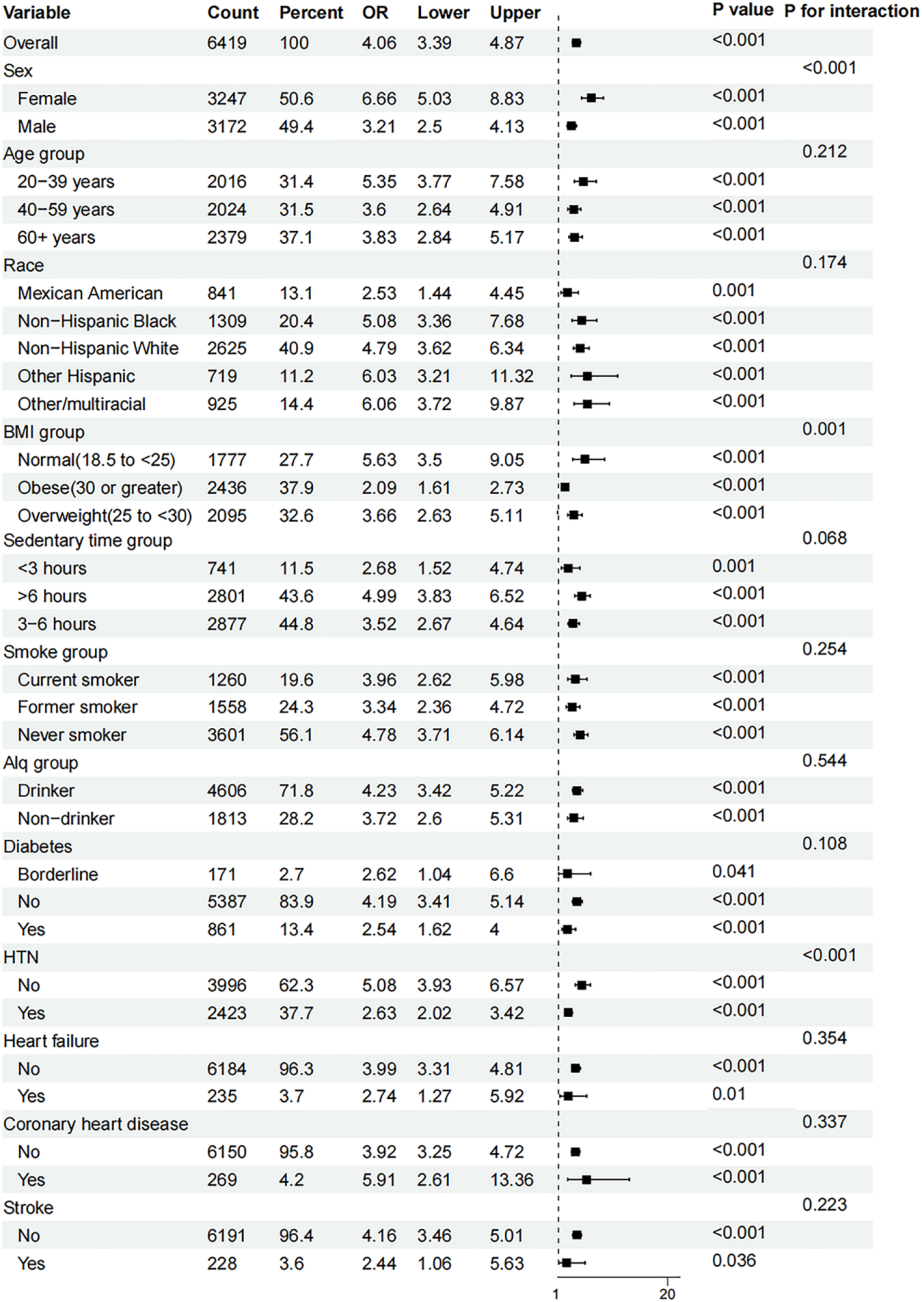
Subgroup analyses of the effect of AIP and hyperuricemia. AIP, atherogenic index of plasma; Alq, alcohol consumption status; BMI, body mass index; OR, odds ratio.

Subgroup analyses (**Figure 4**) based on age, sex, race, BMI, sedentary time, hypertension, smoking status, diabetes, alcohol consumption status, heart failure, coronary heart disease, and stroke demonstrated that the relationship between AIP and hyperuricemia was stable (all *P* < 0.05). However, sex, BMI group and hypertension (*P* for interaction < 0.001, 0.001, and < 0.001, respectively) were considered the most prominent interactive factors influencing the relationship between the AIP and hyperuricemia. With the increase in the AIP, the risk of hyperuricemia among female was more significant than that among male. Participants with normal BMI and no hypertension had a stronger association between AIP and the risk of hyperuricemia than those with BMI greater than 25 kg/m^2^ and hypertension.

## 4 Discussions

In this cross-sectional study including 9439 participants based on NHANES surveys conducted between 2005 and 2018, we found a non-linear, approximately L-shaped dose-response relationship between the AIP and uric acid levels as well as the prevalence of hyperuricemia, with inflection points at 0.588 and 0.573, respectively. On the left side of the inflection points, an increase in AIP was associated with a significant rise in uric acid levels and hyperuricemia prevalence. However, on the right side of the inflection points, there was no significant correlation between AIP and uric acid levels or hyperuricemia prevalence. This indicates that AIP has a saturation effect on uric acid levels and hyperuricemia prevalence once a certain level is reached. Thus, AIP has a saturation effect on both uric acid levels and the prevalence of hyperuricemia.

In this study, AIP was significantly associated with demographic characteristics, lifestyle, and health indicators in adults. With increasing AIP values, parameters such as age, BMI, waist circumference, family poverty income ratio, diabetes, hypertension, coronary heart disease, heart failure, SUA, hyperuricemia,TG, TC, and low-density lipoprotein cholesterol showed an upward trend, while education level and HDL-C levels demonstrated a downward trend. Similar to previous studies, these findings underscore the importance and broad impact of AIP as a risk factor for cardiovascular health (16). AIP, a dependable biological indicator, is linked not only to the development of CVDs, but also to different disorders involving blood lipid metabolism (17–19).

Whether considered as a continuous variable or categorized into quartiles, AIP consistently exhibited a significant positive association with uric acid levels and hyperuricemia, even after controlling for multiple confounding factors. These results demonstrate that AIP remains a significant associator even in multivariate regression analysis, suggesting its potential role as an independent risk factor in the mechanisms of uric acid metabolism and hyperuricemia (9, 20). Hyperuricemia has previously been widely recognized as a risk factor for various chronic diseases, including CVD (2, 21), diabetes (22), and metabolic syndrome (23). This study’s finding of a significant positive relationship between AIP and hyperuricemia deepens our understanding of AIP’s crucial role in metabolic abnormalities. Even when AIP was fully adjusted covariates and categorized into quartiles, the associations remained robust, with a significant trend apparent. These findings are consistent with previous research (10), further validating the positive relationship between high AIP values and hyperuricemia (24).

Interestingly, our results not only clearly illustrated the association between AIP and the prevalence of hyperuricemia as well as uric acid levels, but also showed a saturated, non-linear relationship between AIP and both the prevalence of hyperuricemia and uric acid levels. This finding is significant because it indicates that the impact of AIP intensifies within a specific range before leveling off. Even after adjusting for multiple factors, this non-linear relationship remains. Previous studies have mostly focused on linear or non-linear associations (20), whereas our study further reveals a more complex L-shaped nonlinear relationship between AIP and uric acid levels and hyperuricemia. This suggests that controlling AIP may have a more substantial effect on alleviating hyperuricemia and related metabolic diseases in populations with high AIP. Our study’s examination of the saturation effect of AIP on uric acid levels and hyperuricemia reveals crucial insights that could influence clinical practice and public health strategies. The identification of distinct AIP thresholds, beyond which the correlation with uric acid levels and hyperuricemia becomes non-significant, suggests that there is a limit to how much AIP can impact these parameters. This saturation effect underscores the necessity of targeting individuals with AIP levels below these thresholds for more effective management of uric acid-related conditions. Through systematic analysis, our study revealed a distinct L-shaped relationship and saturation effect between AIP and uric acid metabolism, extending beyond conventional linear correlation analyses. By identifying specific AIP thresholds, we observed clear inflection points in AIP’s influence on uric acid levels, providing new perspectives for clinical practice. These findings suggest that the effectiveness of interventions may vary among populations with different AIP levels, which has potential implications for developing individualized prevention and treatment strategies.

Moreover, our subgroup analyses indicate that the relationship between AIP and hyperuricemia remains consistent across various demographic and health-related variables. Notably, significant interactions with factors such as age, sex, race, and health status (e.g., diabetes and heart failure) highlight the multifaceted nature of AIP’s impact. These findings suggest that while AIP is broadly applicable as a risk marker, its predictive value may be modulated by specific individual characteristics, warranting a personalized approach in clinical interventions (20, 24, 25). The significant interactions with sex, BMI, and hypertension, and the absence of significant interactions with alcohol consumption status, coronary heart disease, and stroke are consistent with previous research (25). This consistency reinforces the robustness of AIP as an important marker for uric acid metabolism disorders.

Extensive evidence has confirmed HDL-C’s protective role against elevated SUA (26, 27). Prior investigations have revealed that the liver’s fatty acid synthesis is associated with the *de novo* purine synthesis process and increased urea generation (28). The increase inTG triggers greater free fatty acid generation, enhancing the dissociation of adenosine triphosphate and culminating in elevated levels of uric acid, the final product of purine metabolic activities (29). Therefore, it is reasonable that AIP, comprisingTG as the numerator and HDL-C as the denominator, is positively linked to the presence of hyperuricemia.

In summary, our results not only advance the understanding of the non-linear dynamics between AIP and uric acid metabolism but also emphasize the importance of strategic thresholds in managing hyperuricemia. These insights pave the way for future research to explore the underlying mechanisms and to develop targeted therapeutic strategies aimed at optimizing AIP and consequently mitigating risks associated with elevated uric acid levels.

Several limitations of this should be acknowledged. First, the cross-sectional design of the study precludes the establishment of causality between AIP and uric acid levels or hyperuricemia risk. Longitudinal studies are necessary to confirm these associations and elucidate potential causal pathways. Second, the reliance on self-reported data for certain variables, such as lifestyle factors and medical history, may introduce recall bias and affect the accuracy of the findings. Third, while we adjusted for a wide range of confounding variables, residual confounding cannot be entirely ruled out. Finally, the study population was derived from the NHANES dataset, which may limit the generalizability of the findings to other populations with different demographic and clinical characteristics.

## 5 Conclusion

In conclusion, our findings indicate a significant L-shaped relationship between AIP and both uric acid levels and hyperuricemia prevalence, with a notable saturation effect beyond specific AIP thresholds. These results suggest that AIP could serve as a valuable marker for identifying individuals at increased risk of elevated uric acid levels and hyperuricemia. However, the observed associations warrant further investigation through prospective studies to confirm causality and explore the underlying mechanisms. Future research should also consider the potential impact of interventions targeting AIP on uric acid metabolism and hyperuricemia risk, which could have important implications for both the prevention and management of related metabolic disorders.

## Data Availability

The original contributions presented in the study are included in the article/supplementary material. Further inquiries can be directed to the corresponding author.
